# I_h_ Channels Control Feedback Regulation from Amacrine Cells to Photoreceptors

**DOI:** 10.1371/journal.pbio.1002115

**Published:** 2015-04-01

**Authors:** Wen Hu, Tingting Wang, Xiao Wang, Junhai Han

**Affiliations:** 1 Institute of Life Sciences, Key Laboratory of Developmental Genes and Human Disease, Southeast University, Nanjing, China; 2 Co-innovation Center of Neuroregeneration, Nantong University, Nantong, China; Stanford University School of Medicine, UNITED STATES

## Abstract

In both vertebrates and invertebrates, photoreceptors’ output is regulated by feedback signals from interneurons that contribute to several important visual functions. Although synaptic feedback regulation of photoreceptors is known to occur in *Drosophila*, many questions about the underlying molecular mechanisms and physiological implementation remain unclear. Here, we systematically investigated these questions using a broad range of experimental methods. We isolated two *I_h_* mutant fly lines that exhibit rhythmic photoreceptor depolarization without light stimulation. We discovered that I_h_ channels regulate glutamate release from amacrine cells by modulating calcium channel activity. Moreover, we showed that the eye-enriched kainate receptor (EKAR) is expressed in photoreceptors and receives the glutamate signal released from amacrine cells. Finally, we presented evidence that amacrine cell feedback regulation helps maintain light sensitivity in ambient light. Our findings suggest plausible molecular underpinnings and physiological effects of feedback regulation from amacrine cells to photoreceptors. These results provide new mechanistic insight into how synaptic feedback regulation can participate in network processing by modulating neural information transfer and circuit excitability.

## Abstract 2

A systematic study of the *Drosophila* visual system clarifies the molecular mechanisms and physiological effects of feedback regulation of photoreceptors by amacrine cells, essential for maintaining light sensitivity.

## Author Summary

Feedback regulation is a common feature of neural circuits during the process of acquiring information. Therefore, it is important to understand how this phenomenon occurs. Using the primary visual system of the fruit fly *Drosophila melanogaster* as a model, we systematically investigated the molecular mechanisms and the physiological implementation of feedback regulation from amacrine cells (second order neurons that are present in the lamina) to photoreceptors. We isolated two fly lines with mutations in the gene that encodes for the ion channel known as I_h_, whose photoreceptors exhibited rhythmic depolarizations in the absence of light stimulation. We demonstrated that I_h_ channels function in amacrine cells to regulate the release of the neurotransmitter glutamate by modulating the activity of the voltage-gated calcium channel, Cac. We further found that the glutamate signal released by amacrine cells is sensed and transduced by glutamate receptors expressed by the photoreceptors. Finally, we showed that this feedback regulation is critical for maintaining light sensitivity in the presence of ambient light. Our results suggest that regulation of synaptic feedback in a neuronal network modulates information transfer and circuit excitability.

## Introduction

Feedback regulation is common in neural circuit information processing. In both vertebrate and invertebrate visual systems, photoreceptor output is feedback-regulated by interneurons, which is an important mechanism for shaping the transmission of light information [1,2]. In the vertebrate retina, bipolar cells receive synaptic input from rod and cone photoreceptors and transfer information to ganglion cells. Meanwhile, the laterally distributed horizontal cells provide a feedback signal to photoreceptor axon terminals, controlling their output gain [3,4]. The structure, function, and development of the vertebrate and insect visual systems possess many evolutionary parallels [5]. In the *Drosophila* lamina, 12 neuron classes have been identified, and specific interneurons may serve similar functions [6,7]. Serial electron-micrograph (EM) studies have revealed that outer photoreceptor (R1–R6) axons project their outputs to L1–L3 monopolar cells and amacrine cells (AC) and receive synaptic inputs from L2, L4, AC, Lawf, and C3 cells [6–8]. Because connectivity in the *Drosophila* lamina has been elucidated to the level of individual synapses, this system provides a good model to study how the feedback neural circuit works and facilitates network information processing [6,7,9,10].

Upon light stimulation, *Drosophila* photoreceptors undergo depolarization via activating the phototransduction cascade, which opens transient receptor potential (TRP) channels [11]. In turn, depolarized photoreceptors release the inhibitory neurotransmitter histamine [12] and hyperpolarize postsynaptic L1–L3 neurons and ACs by opening their histamine-gated chloride channel, HisCl 2 [13]. Intracellular recordings from L1–L3 neurons and R1–R6 photoreceptors imply that L2 and AC receive inhibitory input from R1–R6 axons and subsequently depolarize the photoreceptors through synaptic excitation (glutamate, acetylcholine, or both) [14]. Microinjection, immunolabeling, and genetic reporter line experiments suggest that AC and L2 are either glutamatergic or cholinergic neurons, while L4 is either cholinergic or gabaminergic [12,15–18]. However, the physiological roles of these feedback regulations and the underlying molecular mechanisms remain unclear. In addition, the types of excitatory neurotransmitter receptors in R1–R6 photoreceptors are still unknown.

I_h_ channels, also called hyperpolarization-activated cyclic nucleotide-gated (HCN) channels, are low-threshold, voltage-gated ion channels that are normally activated at negative potentials below −50 mV [19,20]. As I_h_ channels are permeable to Na^+^ and K^+^ and form an inward current at rest, they may depolarize the neuronal resting membrane potential (RMP) and influence excitatory postsynaptic potential kinetics and integration [21–23]. A recent study demonstrated that HCN1 colocalizes with low-threshold voltage-gated T-type Ca^2+^ channels (Ca_v_3.2) in presynaptic terminals and inhibits glutamate release by suppressing Ca_v_3.2 activity [24]. In the present study, we examined two *I*_*h*_ mutant fly lines that exhibit rhythmic depolarization in photoreceptors without light stimulation. Our results demonstrate that I_h_ channels are expressed in ACs and suggest that I_h_ channels regulate synaptic glutamate release by modulating the activity of Cacophony (Cac) channels. We further showed that the eye-enriched kainate receptor (EKAR) receives the retrograde glutamate signal in photoreceptor terminals. Finally, we investigated how feedback regulation from ACs affects photoreceptor output and fly optomotor behavior. Our studies elucidate the molecular mechanism and physiological roles of feedback regulation from ACs to photoreceptors in the *Drosophila* visual system.

## Results

### *I*_*h*_ Mutant Photoreceptors Undergo Rhythmic Depolarization

**Fig 1:**
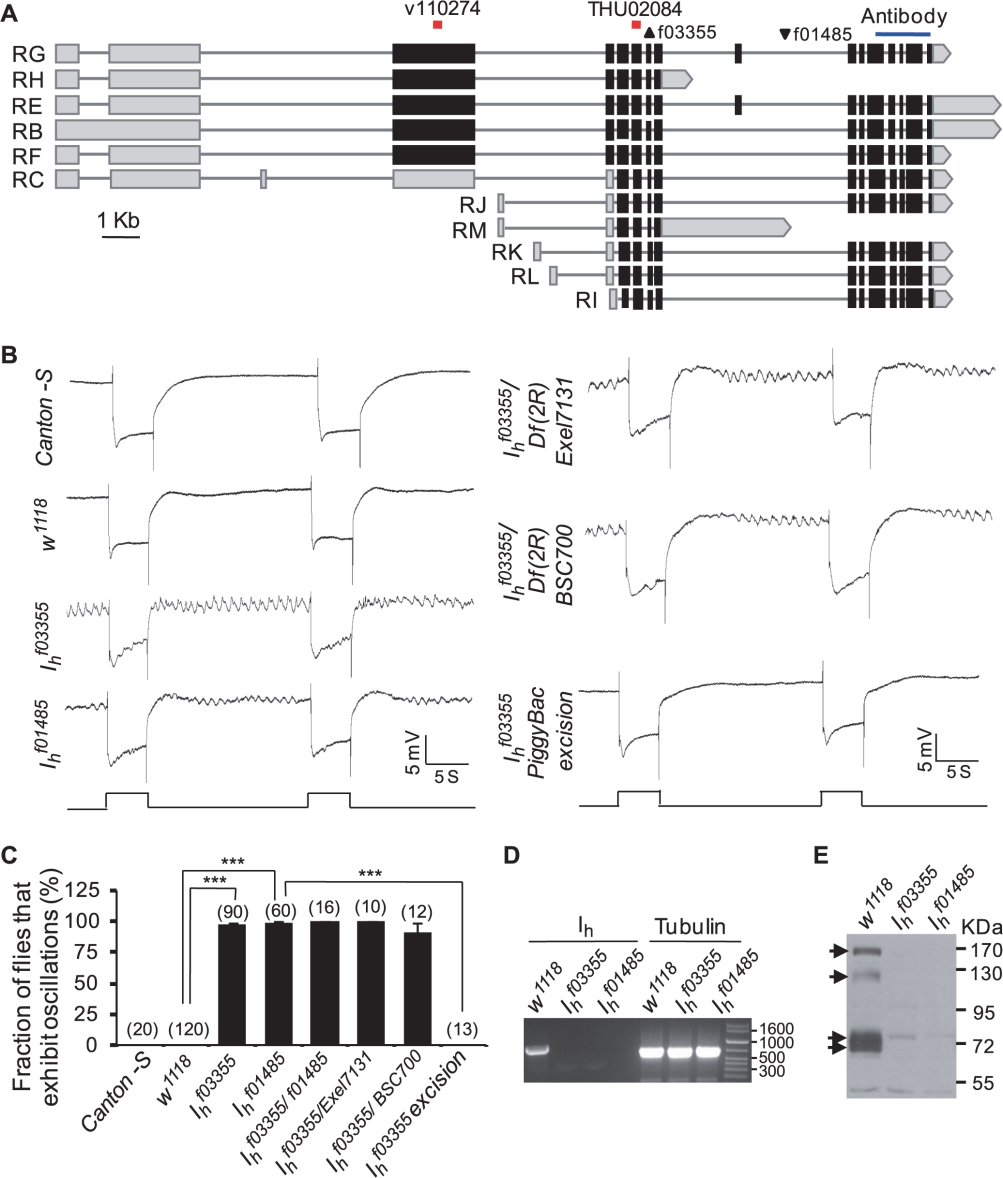
*I*_h_ mutant lines exhibit ERG baseline oscillation. (**A**) Annotated transcriptions of the *I*_*h*_ gene. Two *piggyBac* insertion sites are marked with triangles. The RNAi recognized site and coding region used for antibody generation are labeled at the top. (**B**) *I*_*h*_ mutant lines exhibit ERG baseline oscillation. For ERG traces throughout all figures, event markers represent 5-s orange light pulses, and scale bars are 5 mV. (**C**) Fraction of flies that exhibit the ERG oscillation phenotype in each genotype. The numbers of recorded flies for each genotype are listed. (**D**) RT-PCR shows *I*_*h*_ mRNAs are transcripted in wild-type flies but are absent in *I*_*h*_ mutant flies. Primer pair CACGCGACCAATCTCATCC/ TCATGGAGTGTTACCCTCG, which can amplify all transcriptional variants, was used in RT-PCR analysis. The tubulin gene was used as a loading control. (**E**) Western blotting revealed four major I_h_ channel variants (indicated by arrows) expressed in wild-type flies but absent in *I*_*h*_ mutant flies. Note that the low-intensity bands presented in *I*_*h*_ mutant flies are nonspecific.

To identify additional genes involved in fly photoreceptor functions, we performed an electroretinogram (ERG)-based genetic screen in the mutant lines from Exelexis collections [25,26] and gene disruption project (GDP) collections [27]. In this screen, we identified two *I*_*h*_ mutant lines, *PBac{XP}I*_*h*_^*f01485*^ and *PBac{XP}I*_*h*_^*f03355*^, that exhibited normal light responses and distinctive ERG baseline oscillations (Fig. 1A–C). Although the amplitudes and frequencies of oscillations were variable within individual flies (S1A Fig.), this ERG phenotype was easily detectable and distinct from that of wild-type flies, which never exhibited ERG baseline oscillations (Fig. 1B,C). Strikingly, ERG baseline oscillations in *I*_*h*_ mutant flies were sustained for more than 15 min, although their amplitude and frequency were attenuated (S1B Fig.). Similar results were observed in the recombinants with two deficiency lines *Df(2R)Exel7131* and *Df(2R)BSC700*, in which the entire *I*_*h*_ gene (Gene ID: 36589) was deleted (Fig. 1B,C) [26,28]. Splicing of the *I*_*h*_ gene creates several transcriptional variants that encode I_h_ channels with long or short N-termini and different lengths of the inter-loop regions between the membrane-spanning domains S3–S4 and S4–S5 (Fig. 1A) [29]. The *PBac{XP}I*_*h*_^*f03355*^ mutant contains a piggyBac inserted into the extron of all transcriptional variants of the *I*_*h*_ gene, whereas *PBac{XP}I*_*h*_^*f01485*^ mutant has a *piggyBac* inserted into the intron of most transcriptional variants of the *I*_*h*_ gene (Fig. 1A) [25]. Reverse transcription polymerase chain reaction (RT-PCR) analysis revealed that the *piggyBac* insertion completely abolished the mRNA transcription of I_h_ gene in these two mutant lines (Fig. 1D). Using antibodies against the intracellular C terminal domain that exists in all I_h_ channel variants, we revealed four major I_h_ channel variants (170, 125, 73, and 71 kDa) that were expressed in wild-type flies but absent in *I*_*h*_ mutant flies (Fig. 1E). In addition, two low intensity bands (74 and 52 kDa) exist in *I*_*h*_ mutants (Fig. 1E), which might be nonspecific bands. To further confirm that *piggyBac* insertion actually disrupts the I_h_ gene and leads to ERG baseline oscillations in *PBac{XP}I*_*h*_^*f03355*^ mutants, we performed *piggyBac* precise excision from *PBac{XP}I*_*h*_^*f03355*^ mutants. ERG recording showed that this completely abolished the ERG baseline oscillations phenotype (Fig. 1B,C). Taken together, these results demonstrate that loss of I_h_ channels results in an abnormal ERG baseline oscillation phenotype.

**Fig 2:**
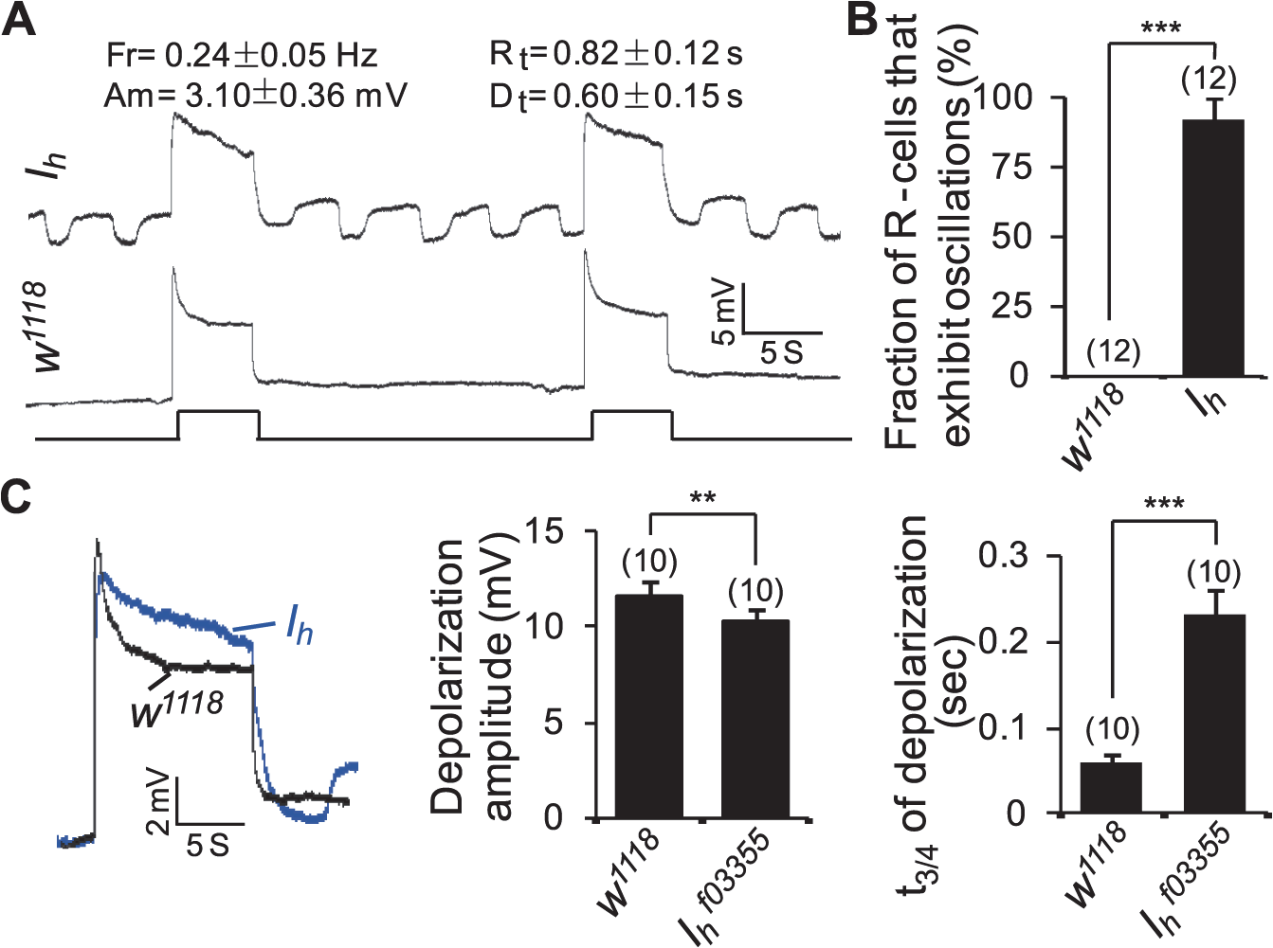
*I*_h_ mutant photoreceptors undergo rhythmic depolarization without light stimulation. (**A**) Intracellular recording traces of wild-type and *I*_*h*_ mutant photoreceptors. For intracellular recording traces, event markers represent 5-s orange light pulses, and scale bars are 5 mV. Measurements of the amplitude (Am), frequency (Fr), rise time (R_t_), and decay time (D_t_) of rhythmic depolarization are provided at the top. (**B**) The fraction of photoreceptors (R-cells) that exhibit oscillation phenotype. The numbers of photoreceptors recorded for each genotype are listed. (**C**) Measurement of the amplitude of light-induced depolarization (middle) and the time (t_3/4_) required for a 3/4 recovery from the responses upon stimulation cessation (right). *n* = 10.

Because ERG is an extracellular recording technique that measures the light-induced mass response of the eye, we next conducted intracellular recordings in photoreceptors to investigate whether the ERG baseline oscillation phenotype was due to a photoreceptor abnormality. Interestingly, *I*_*h*_ mutant photoreceptors, but not wild-type photoreceptors, showed rhythmic depolarization without light stimulation (Fig. 2A,B). In the first several minutes of recording, the pacemaker traces in *I*_*h*_ mutant flies showed a uniform amplitude (3.10 ± 0.36 mV) and frequency (0.24 ± 0.05 Hz). Each depolarization had a rise time of 0.82 ± 0.12 s, lasted 1.57 ± 0.13 s, and had a decay time of 0.60 ± 0.15 s (Fig. 2A). The frequency of depolarization in the intracellular recordings was much lower than that in ERG recordings, likely due to the desynchronized depolarization of multiple photoreceptors in the extracellular recordings. In addition, *I*_*h*_ mutant photoreceptors showed a reduced amplitude (10.3 ± 0.7 mV versus 11.6 ± 0.8 mV, *p* < 0.01) as well as a prolonged decay time (0.23 ± 0.03 s versus 0.06 ± 0.01 s, *p* < 0.001) of light-evoked depolarization (Fig. 2C). These alterations might reflect abnormal photoreceptors and/or cell communication in the visual system.

**Fig 3:**
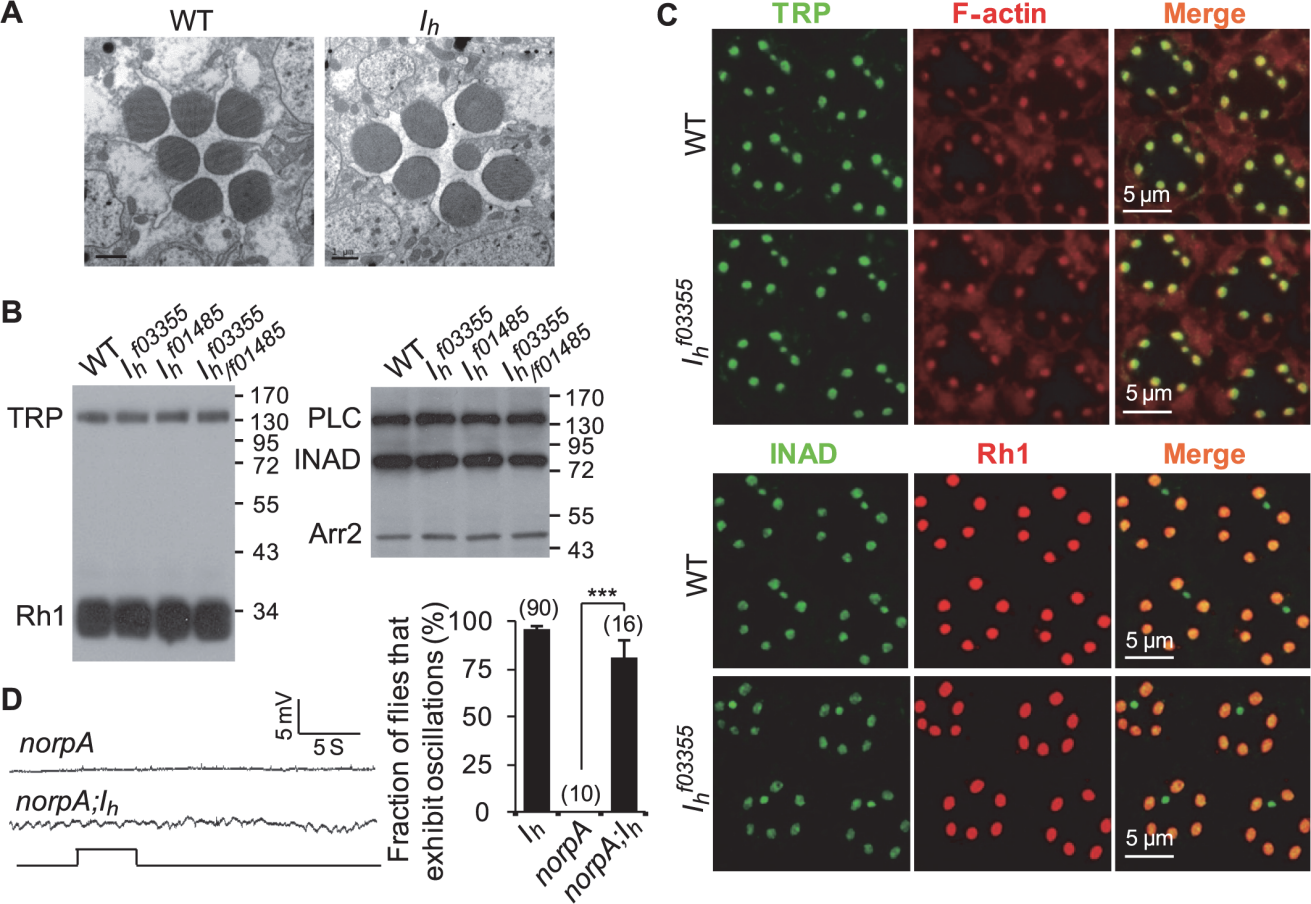
*I*_h_ mutant flies show normal rhabdomeral structure, normal protein levels, and normal distribution of phototransduction components. (**A**) EM images show normal rhabdomeral structure in 1-day-old *I*_*h*_ mutant flies. (**B**) Western blotting shows normal protein levels of phototransduction components in *I*_*h*_ mutant flies. (**C**) Immunostaining images show normal distribution of phototransduction components in *I*_*h*_ mutant flies. (**D**) ERG traces of *norpA* and *norpA;I*_*h*_ flies. The fraction of flies that exhibit ERG oscillation phenotype are shown in the right panel, and the number of recorded flies for each genotype are listed. WT = wild type.

To explore whether loss of I_h_ channels causes a photoreceptor abnormality, we conducted EM studies and biochemical analyses of photoreceptors. EM images showed normal rhabdomeral structures in *I*_*h*_ mutant flies (Fig. 3A), and the expression and localization of phototransduction components in *I*_*h*_ mutant flies were also normal (Fig. 3B,C), indicating that the rhythmic depolarization noted in *I*_*h*_ mutant photoreceptors was not due to abnormalities in rhabdomere structure or phototransduction cascades. We also excluded the possibility that the rhythmic depolarization in *I*_*h*_ mutant photoreceptors was dependent on phototransduction cascades activation by genetically blocking phototransduction cascade activation through introducing the mutant of *norpA*, which encodes the key phototransduction component phospholipase C [30]. ERG recordings showed that *norpA* mutation did not suppress the rhythmical depolarization in *I*_*h*_ mutant photoreceptors (Fig. 3D).

### Lack of I_h_ Channels in ACs Leads to Rhythmic Depolarization in Photoreceptors

**Fig 4:**
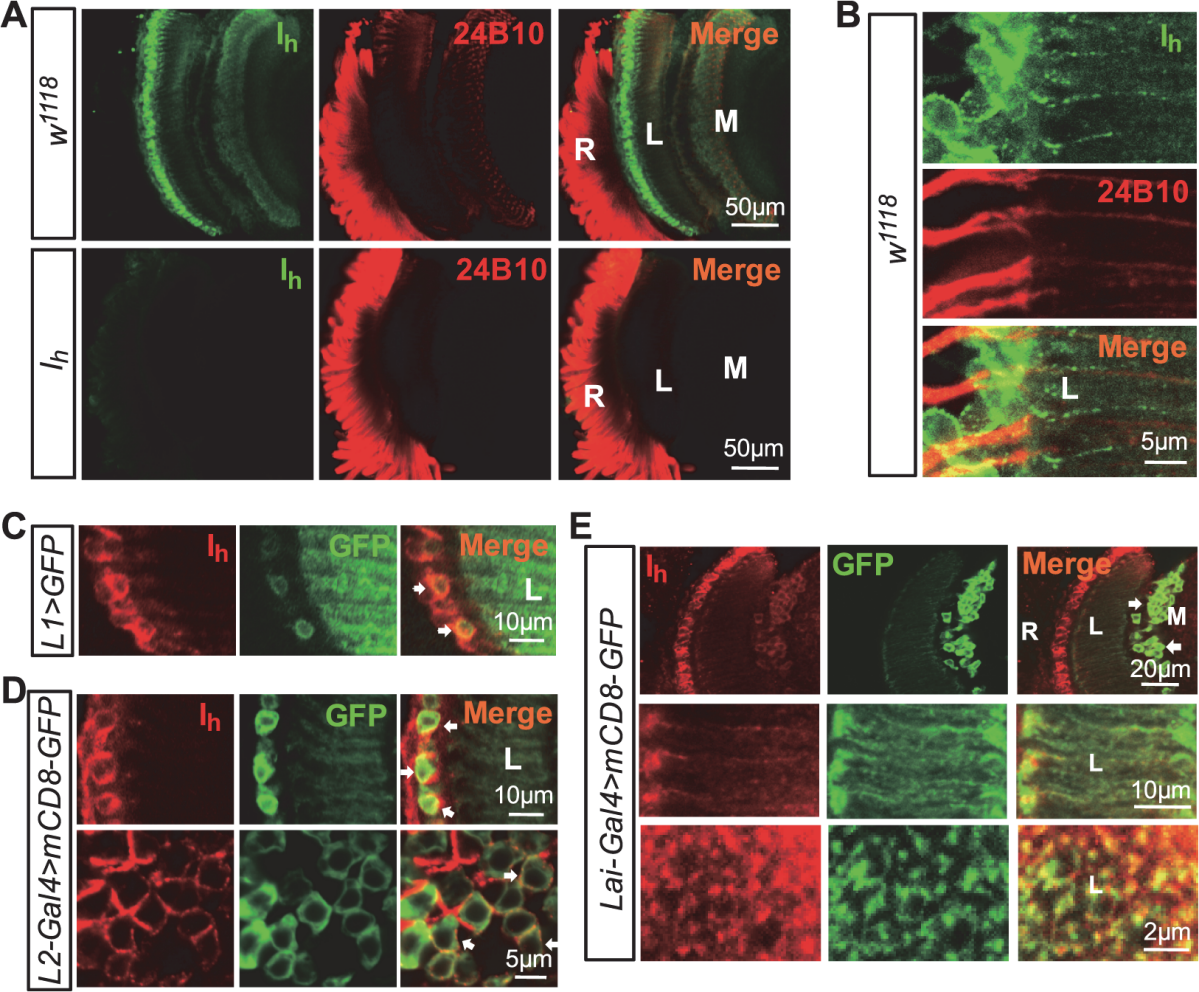
Expression patterns of endogenous I_h_ channels. (**A**) Localization of endogenous I_h_ channels in the adult fly head. Dissected whole heads were double stained with anti-I_h_ (green) and 24B10 (red, for photoreceptor membrane) antibodies. The images show a longitudinal view of the retina (R), lamina (L), and medulla (M). (**B**) Distribution of I_h_ channels in the lamina region. Images show a longitudinal view. (**C**) I_h_ channels expressed in L1 neurons. L1 neurons were labeled with mCD8-GFP under the control of the *L1-GAL4* driver. Two L1 somata are indicated by arrows. (**D**). I_h_ channels were highly expressed in L2 neurons. L2 neurons were labeled with mCD8-GFP under the control of the *L2-GAL4* driver. The upper panel shows a longitudinal view of the lamina, and the lower panel shows a cross view of the lamina. L2 somas are indicated by arrows. (**E**) I_h_ channels were expressed in ACs. ACs (arrows) are labeled with mCD8-GFP under the control of the *Lai-GAL4* driver. The upper panel shows a longitudinal view of the retina (R), lamina (L) and medulla (M), and the middle and lower panels show a longitudinal and cross-sectional view of AC processes.

To elucidate how the loss of I_h_ channels leads to rhythmic depolarization in photoreceptors, we examined the expression pattern of endogenous I_h_ channels. In wild-type flies, I_h_ channel staining was observed in the lamina and medulla, whereas photoreceptor cell bodies were either weakly or not at all labeled (Fig. 4A). Although the axons of outer photoreceptors project into the lamina and form synaptic connections with multiple lamina neurons, I_h_ channels were undetectable in photoreceptor axons (Fig. 4B). Strong I_h_ labeling was observed in the somata of L1 and L2 neurons, whose membranes had been labeled with mCD8-GFP markers (Fig. 4C,D). I_h_ channels were also found in the somata and processes of ACs, which was identified by expression of mCD8-GFP markers under the control of AC-specific split GAL4 (Lai-GAL4) (Fig. 4E) [31].

**Fig 5:**
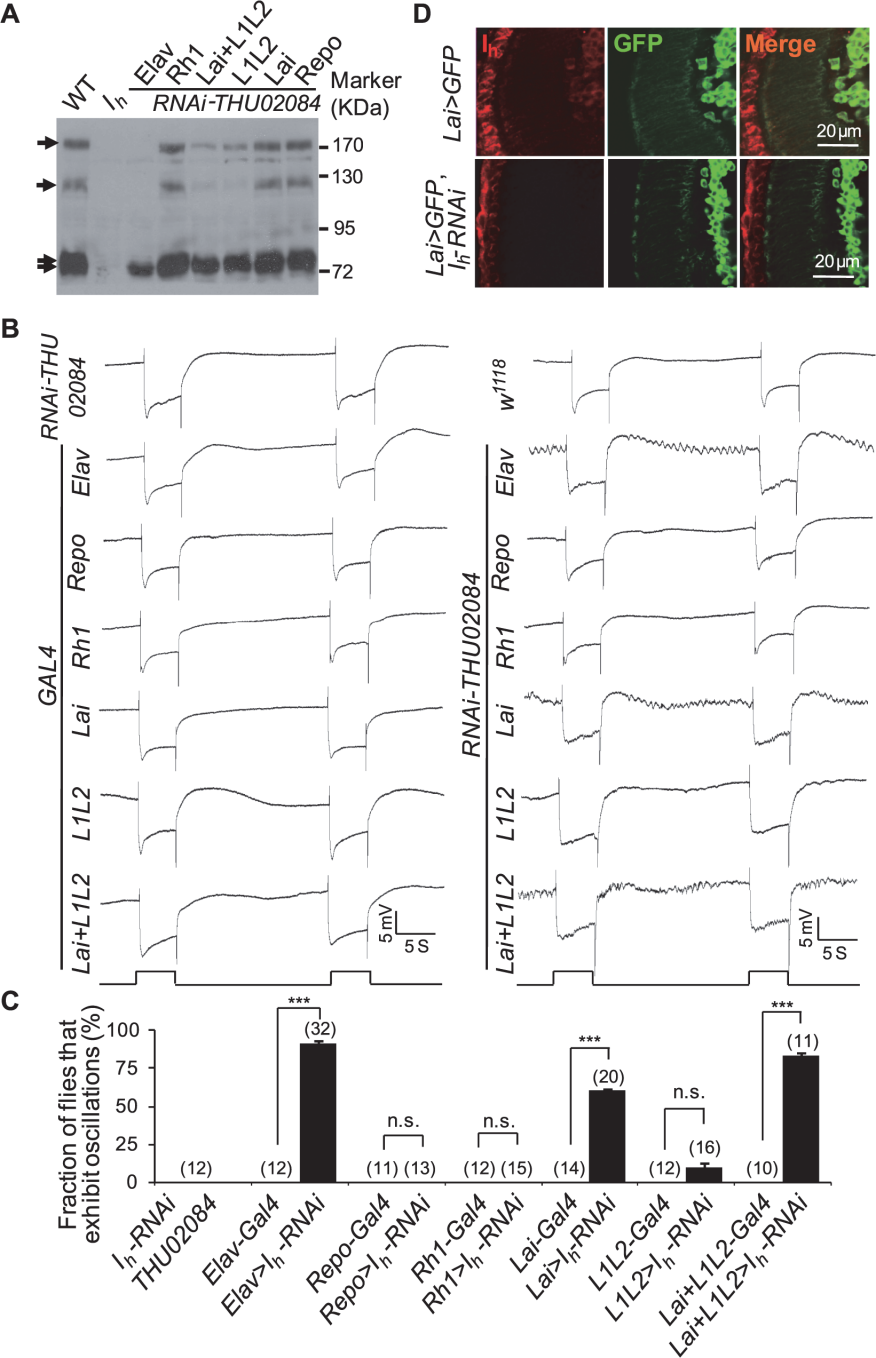
Depletion of I_h_ channels in ACs results in rhythmic depolarization in photoreceptors. (**A**) I_h_ channel expression levels in flies with I_h_ channel depletion using *UAS-I*_*h*_-*RNAi* driven by anatomically restricted GAL4 drivers. A single copy of the GAL4 driver was used for each GAL4 line. Each lane was loaded with two fly heads. The I_h_ channel bands are indicated with arrows. (**B**) ERG traces of flies with I_h_ channels depletion using *UAS-I*_*h*_-*RNAi* driven by anatomically restricted GAL4 drivers. A single copy of the GAL4 driver was used for each GAL4 line. (**C**) The fraction of flies that exhibit ERG oscillation phenotype in each genotype. The numbers of recorded flies for each genotype are listed. (**D**) Expression of I_h_ channels in *UAS-mCD8-GFP*,*UAS-I*_*h*_-*RNAi/Lai-Gal4* (bottom) and control (top) flies. Dissected whole brains were stained with anti-I_h_ (red) and anti-GFP (green) antibodies. Note that I_h_ channel distribution in *UAS-mCD8-GFP*,*UAS-I*_*h*_-*RNAi/Lai-Gal4* flies is comparable to control flies except in ACs.

To determine whether I_h_ channels in the lamina neurons or glia might contribute to rhythmic depolarization in photoreceptors, we specifically depleted I_h_ channels in various cell types using an RNAi knockdown approach. The RNAi line THU02084.N recognizes all *I*_*h*_ transcriptional variants (Fig. 1A) [32], and western blotting analysis demonstrated that RNAi knockdown of I_h_ channels using pan-neural *elav-GAL4* but not glia-specific *repo-GAL4* successfully repressed the expression of all I_h_ variants (Fig. 5A). Moreover, RNAi against all *I*_*h*_ transcriptional variants using *elav-GAL4* but not *repo-GAL4* phenocopied the abnormal ERG baseline oscillations observed in *I*_*h*_ mutant flies (Fig. 5B,C), indicating that the loss of I_h_ channels in neurons, but not glia, results in rhythmic depolarization in photoreceptors. Consistent with no or low I_h_ channel expression in photoreceptors, the *Rh1-GAL4* driver did not recapitulate the ERG deficits observed in *I*_*h*_ mutant flies (Fig. 5B,C). These results suggest that rhythmical depolarization in *I*_*h*_ mutant photoreceptors is caused by abnormal communication between photoreceptors and other neurons.

Photoreceptor terminals receive synaptic inputs from L2, L4, and Lawf neurons and ACs [6–8]. Given that I_h_ channels are expressed in L1 andL2 neurons and ACs, we next asked whether the loss of I_h_ channels in these neurons causes rhythmic depolarization in photoreceptors. Although RNAi knockdown of I_h_ channels in L1 and L2 neurons using *L1L2-GAL4* [33,34] significantly reduced the levels of I_h_ channels (Fig. 5A), these RNAi knockdown flies did not exhibit obvious ERG baseline oscillations (Fig. 5B,C), suggesting that the loss of I_h_ channels in L1 and L2 neurons is not sufficient to trigger rhythmic depolarization in photoreceptors. Our immunostaining data from *Lai-GAL4;UAS-mCD8-GFP* flies have shown that the number of ACs is small (Fig. 4E), and anti-I_h_ antibody staining revealed that I_h_ expression in ACs is much lower than that in L1/L2 neurons (Fig. 4E). Therefore, depletion of I_h_ channel from ACs may not lead to a detectable reduction in total I_h_ channels in the whole fly head (Fig. 5A). To validate that RNAi knockdown in ACs works well, we performed immnuostaining analysis and showed that RNAi knockdown of I_h_ channels using *Lai-GAL4* successfully did deplete I_h_ channels in ACs (Fig. 5D). Interestingly, RNAi knockdown of I_h_ channels using *Lai-GAL4* recapitulated the ERG abnormalities observed in *I*_*h*_ mutant flies (Fig. 5B,C), indicating that the loss of I_h_ channels in ACs leads to rhythmic depolarization in photoreceptors. Although knockdown of I_h_ channels in L1 and L2 neurons using L1L2-GAL4 caused occasional ERG baseline oscillations, the recombinant of Lai-GAL4 and L1L2-GAL4 resulted in greater ERG baseline oscillations compared with those caused by Lai-GAL4 alone (Fig. 5B,C). These results suggest that the loss of I_h_ channels in L1 and L2 neurons might also contribute to rhythmic depolarization in photoreceptors.

**Fig 6:**
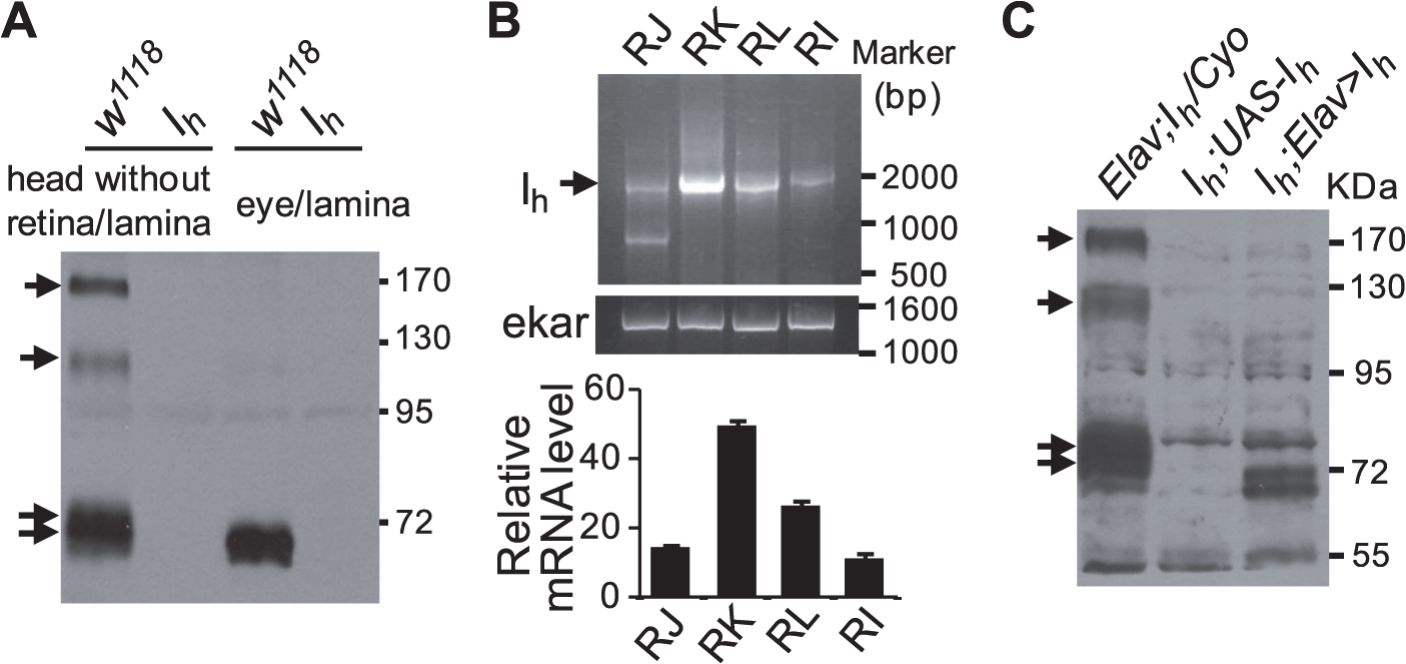
Transcriptional and translational profiles of I_h_ channels. (**A**) Western blotting shows the I_h_ variants expressed in isolated retina/lamina and head without retina/lamina. Each lane was loaded with retina/lamina or head without retina/lamina from two flies. The I_h_ channel bands are indicated with arrows. (**B**) RT-PCR shows the transcriptional profile of I_h_ channels (arrow) in the isolated retina/lamina. Primer pairs used to amplify each transcriptional variants are *I*_*h*_-RJ: GGCACCGCTTGTCACTGCTC/GGATCGAAAGTTGGAGCG; *I*_*h*_-RI: GGCACCGCTTGTCACTGCTC/CTAGACCAGGACAGACAGAC; *I*_*h*_-RL: GGCACCGCTTGTCACTGCTC/GCACGCTTCCAGACTTCTACG; *I*_*h*_-RK: GGCACCGCTTGTCACTGCTC/GCCAGCCAATTTCGGAAGCG. Quantification of relative transcriptional variants of the *I*_*h*_ gene in the isolated retina/lamina is shown at the bottom. The ekar gene was used as a loading control. (**C**) Expression level of I_h_ channels in rescue flies. A single copy of the GAL4 driver was used. The I_h_ channel bands are indicated with arrows.

We next generated *p[UAS-I*_*h*_] transgenic flies and performed rescue experiments. To choose an appropriate variant for transgene generation, we first determined which variant is expressed in the retina and lamina. The RNAi line *P{KK100190}VIE-260B* only recognizes the long isoforms of *I*_*h*_ gene (Fig. 1A) [35]. RNAi against long *I*_*h*_ transcriptional variants using eye-specific *eyeless-GAL4* showed normal I_h_ protein levels and ERG response (S2A,B Fig.). Furthermore, pan-neural *elav-GAL4* failed to recapture the ERG baseline oscillations (S2B,C Fig.), although it caused a significant reduction in the long isoforms of I_h_ proteins (170 and 125 kDa, S2A Fig.). The above observations indicated that only short isoforms of I_h_ play essential roles in suppressing the ERG baseline oscillation phenotype. Western blotting also revealed that 71- and 73-kDa variants but not 170- and 125-kDa variants were highly expressed in the isolated retina and lamina (Fig. 6A). RT-PCR analysis further showed that the annotated transcript form I_h_-RK, which encodes a 618aa isoform I_h_-PK protein, was abundant in the retina and lamina (Fig. 6B). Thus, we amplified the cDNA of I_h_-RK and generated *p[UAS-I*_*h*_^*K*^] transgenic flies to confirm that this transgene can be successfully expressed in neurons (Fig. 6C).

**Fig 7:**
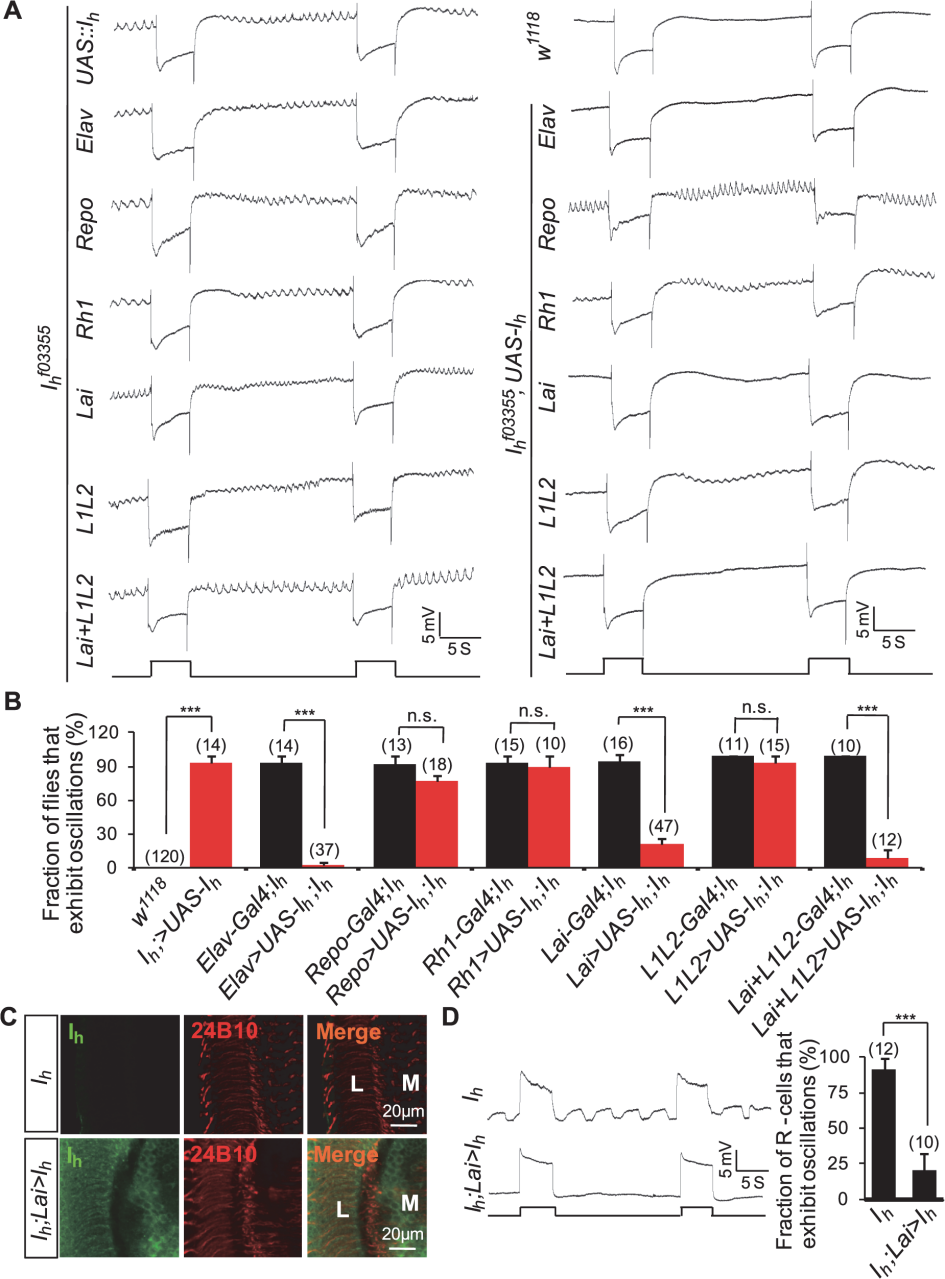
Expression of I_h_ channels in ACs restores a normal ERG response. (**A**) Expression of I_h_ channels in ACs suppresses ERG baseline oscillation. I_h_ channels were expressed using anatomically restricted GAL4 drivers. Flies possessed one copy of the indicated drivers. (**B**) The fraction of flies that exhibit the ERG oscillation phenotype in each genotype. The number of recorded flies for each genotype is listed. (**C**) Expression of I_h_ channels in *I*_*h*_ mutant (top) and *I*_*h*_;*Lai-GAL4/UAS—I*_*h*_ (bottom) flies. Dissected whole brains were stained with anti-I_h_ (green) and anti-24B10 (red) antibodies. L, lamina; M, medulla. (**D**) Intracellular recordings of photoreceptors show that I_h_ channels expression in ACs suppresses rhythmical depolarization without light stimulation. The fractions of photoreceptors that exhibit rhythmic depolarization are presented in the right panel, and the numbers of recorded photoreceptors for each genotype are listed.

Expression of I_h_ channels in ACs but not in L1 and L2 neurons restored normal ERG activity in *I*_*h*_ mutant flies (Fig. 7A–C). Conversely, expression of I_h_ channels in photoreceptors (*Rh1-GAL4*) or glia (*repo-GAL4*) had no inhibitory effects on ERG baseline oscillations (Fig. 7A,B). Intracellular recordings further validated that the expression of I_h_ channels in ACs suppressed rhythmic depolarization in photoreceptors (Fig. 7D). These observations provide evidence that AC-derived I_h_ channels are critical to inhibit rhythmic depolarization in photoreceptors.

### Rhythmic Depolarization in *I*_*h*_ Mutant Photoreceptors Is Due to Uncontrolled Synaptic Glutamate Release from ACs

**Fig 8:**
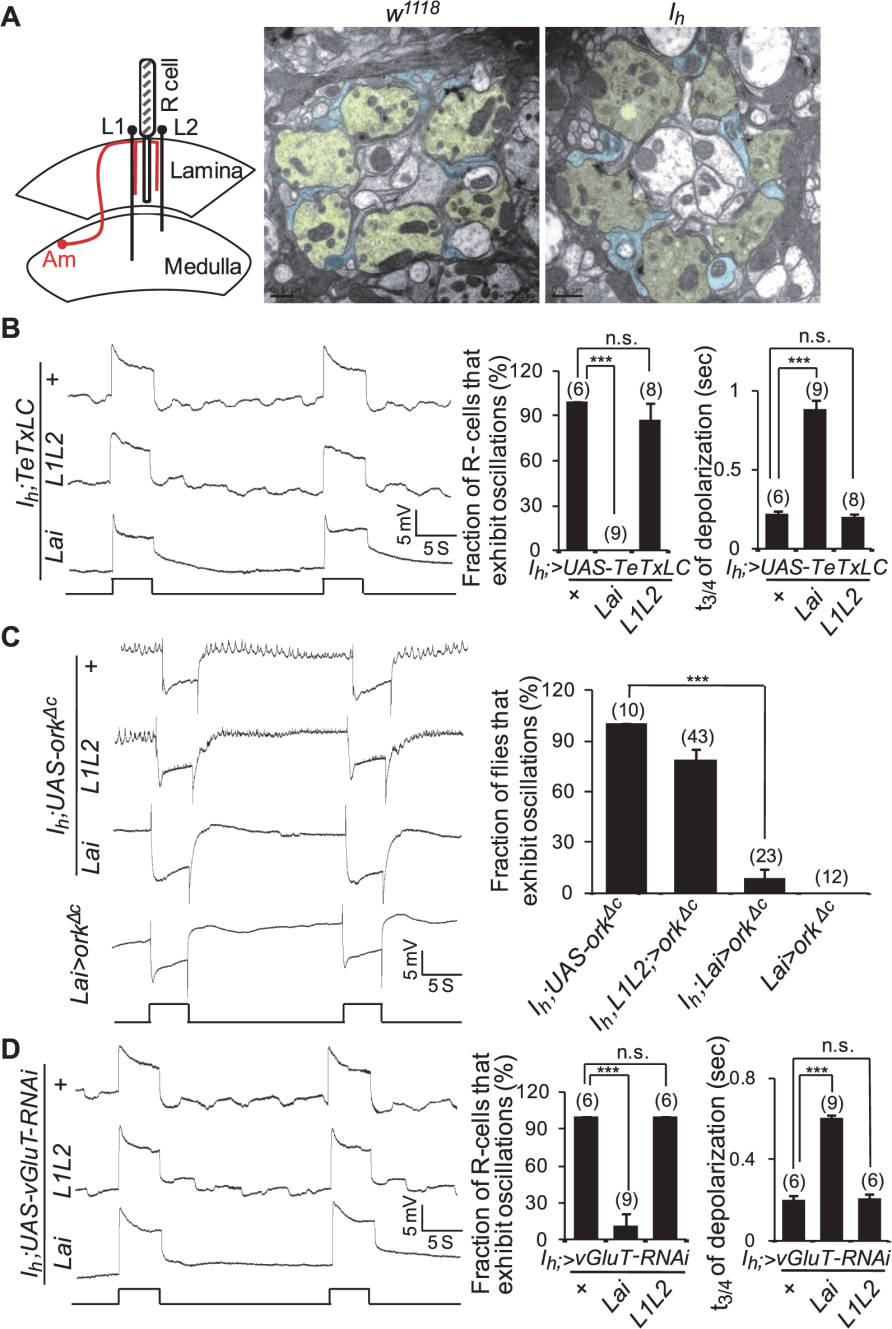
Blocking synaptic glutamate release from ACs suppresses the rhythmic depolarization in *I*_h_ mutant photoreceptors. (**A**) Ultrastructure of lamina cross-sections in wild-type and *I*_*h*_ mutant flies. The left panel shows the organization of the columnar neurons with synaptic connections in the lamina. Photoreceptor cells are shown in gray, L1–L2 neurons in black, and ACs in red. These neurons are present in all lamina columns, and single example profiles are shown arrayed across the lamina. The middle and the right panels show EM images of lamina cross-sections in wild-type and *I*_*h*_ mutant flies, respectively. Photoreceptor axons are colored in yellow and AC processes in blue. (**B**) Intracellular recording traces of *I*_*h*_ mutant flies with expression of TeTxLC using *L1L2-GAL4* and *Lai-GAL4* drivers. The fractions of photoreceptors that exhibit rhythmic depolarization are presented in the middle panel, and the time (t_3/4_) required for a 3/4 recovery from the responses upon stimulation cessation is shown in the right panel. The numbers of recorded flies are listed. (**C**) Inactivation of ACs via ectopic expression of dORK^ΔC^ suppresses rhythmical depolarization in *I*_*h*_ mutant flies. The fractions of flies that exhibit ERG oscillation phenotype and the numbers of recorded flies are presented in the right panel. An ERG trace of flies expressing dORK^ΔC^ in wild-type ACs is also presented. (**D**) Intracellular recording traces of *I*_*h*_ mutant flies expressing *UAS-vGluT-RNAi* using different drivers. The fractions of photoreceptors that exhibit rhythmical depolarization are presented in the middle panel, and the time (t_3/4_) required for a 3/4 recovery from the responses upon stimulation cessation are shown in the right panel. The number of recorded flies for each genotype is listed.

As photoreceptor terminals receive synaptic inputs directly from ACs, we first investigated whether rhythmic depolarization in *I*_*h*_ mutant photoreceptors was due to abnormal cartridge structure or connections between photoreceptors and ACs. However, EM images showed no obvious morphological differences between *I*_*h*_ mutant and wild-type cartridges (Fig. 8A). Therefore, we suspected that rhythmic depolarization in photoreceptors might be due to abnormal synaptic output from ACs. To test this hypothesis, we blocked neurotransmitter release from ACs by expressing tetanus toxin light chain (TeTxLC) [36] or silencing ACs by expressing a mutant form of open rectifier potassium channel (dORK^ΔC^) [37]. TeTxLC and dORK^ΔC^ expression was suppressed during development by the ubiquitous expression of temperature-sensitive Gal80^ts^ but selectively induced during adulthood by exposure to 30°C for 4 h [38]. Interestingly, intracellular recordings revealed that TeTxLC expression in ACs abolished rhythmic depolarization in *I*_*h*_ mutant photoreceptors and led to a prolonged decay time of light-induced depolarization (Fig. 8B and S3A Fig.). By contrast, TeTxLC expression in L1 and L2 neurons failed to suppress rhythmic depolarization in *I*_*h*_ mutant photoreceptors (Fig. 8B and S3A Fig.). Consistently, ectopic expression of dORK^ΔC^ in ACs but not in L1L2 neurons also suppressed rhythmic depolarization in *I*_*h*_ mutant photoreceptors and caused a prolonged decay time of light-induced depolarization (Fig. 8C). These observations indicate that rhythmic depolarization in *I*_*h*_ mutant photoreceptors is due to abnormal synaptic output from ACs.

Given that ACs are likely glutamatergic neurons [16], we further tested whether rhythmic depolarization in photoreceptors is caused by uncontrolled glutamate release from ACs. Vesicular glutamate transporter (vGluT) functions in loading glutamate into synaptic vesicles and is therefore critical for synaptic glutamate output [16]. Consistently, knockdown of vGluT expression in ACs but not in L1 and L2 neurons suppressed rhythmic depolarization in *I*_*h*_ mutant photoreceptors (Fig. 8D and S3B Fig.). Taken together, the above observations demonstrate that rhythmic depolarization in *I*_*h*_ mutant photoreceptors is due to uncontrolled synaptic glutamate release from ACs.

### I_h_ Channels Regulate Glutamate Release from ACs by Limiting Cac Channel Activity

**Fig 9:**
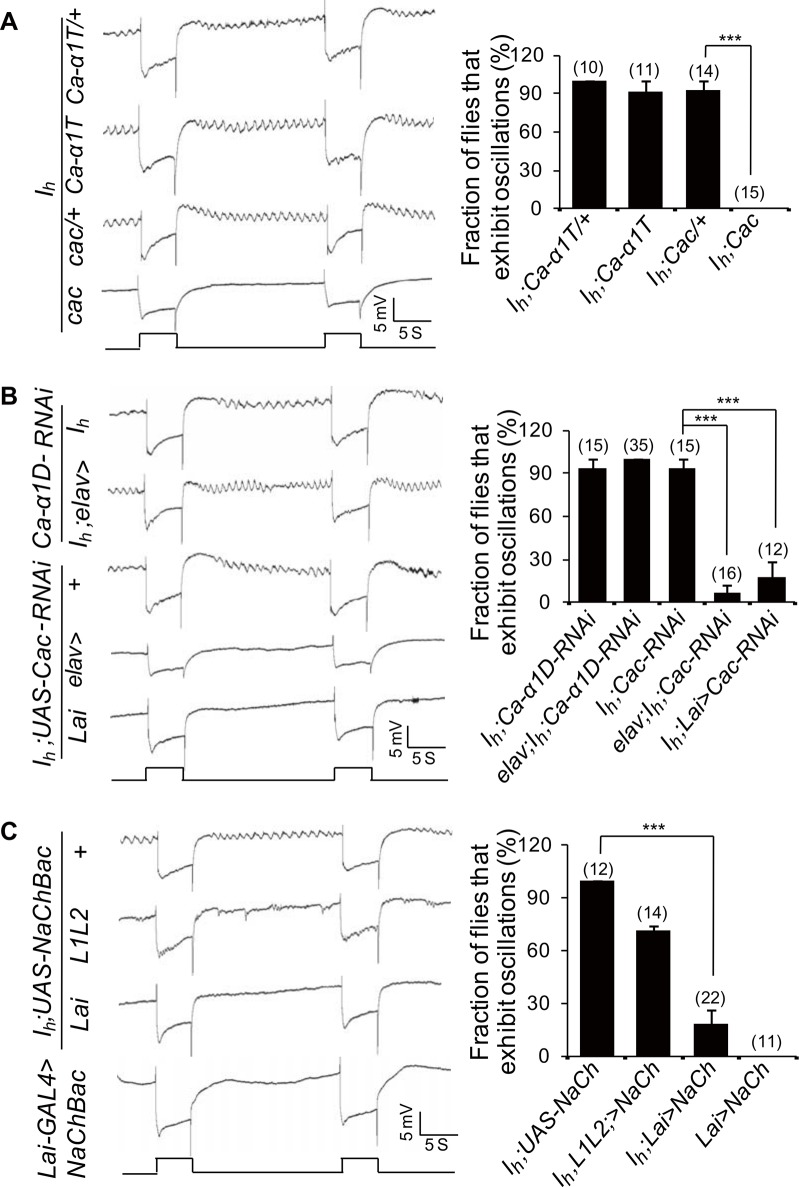
I_h_ channels regulate synaptic glutamate release by modulating Cac channel activity. (**A**) ERG traces of *Ca-α1T; I*_*h*_ and *cac; I*_*h*_ flies. The fractions of flies exhibiting the ERG oscillation phenotype and the number of recorded flies are presented in the right panel. (**B**) ERG traces of flies with depletion of Ca-α1D or Cac channels. The fraction of flies exhibiting the ERG oscillation phenotype and the number of recorded flies are presented in the right panel. (**C**) Depolarization of the RMP of ACs via ectopic expression of NaChBac suppresses rhythmical depolarization in *I*_*h*_ mutant flies. The fractions of flies exhibiting the ERG oscillation phenotype and the number of recorded flies are presented in the right panel. An ERG trace of flies expressing NaChBac in wild-type ACs is also shown.

Previous studies suggest that synaptic release may depend on Ca^2+^ entry via voltage-gated Ca^2+^ channels (VGCCs) [39–41]. Thus, we attempted to identify which VGCC contributes to the mediation of retrograde glutamate release from ACs. The *Drosophila* genome contains three putative homologs of vertebrate VGCCs (Ca-*α*1D, Gene ID: 34950; Cac, Gene ID: 32158; and Ca-*α*1T, Gene ID: 31550) [20]. Interestingly, introducing the *cac* mutant but not the *Ca-α1T* mutation suppressed ERG baseline oscillations in *I*_*h*_ mutant flies (Fig. 9A). In addition, RNAi against *cac* (*elva-GAL4/+;I*_*h*_;*UAS-cac-RNAi/+*) but not *Ca-α1D* (*elva-GAL4/+;I*_*h*_;*UAS-Ca-α1D-RNAi/+*) suppressed ERG baseline oscillations in *I*_*h*_ mutant flies (Fig. 9B). Furthermore, RNAi against *cac* in ACs was also able to suppress ERG baseline oscillations in *I*_*h*_ mutant flies (*I*_*h*_; *Lai-GAL4/UAS-cac-RNAi*) (Fig. 9B). These results suggest that Cac channels mediate synaptic glutamate release from ACs.

Cac channels can produce high voltage-activated Ca^2+^ currents above −30 mV and low voltage-activated Ca^2+^ currents between −70 and −60 mV in vivo [42]. I_h_ channels are normally open at potentials more negative than −50 mV, leading to depolarization of the RMP. A recent study demonstrated that HCN1 colocalizes with Ca_v_3.2 in presynaptic terminals and inhibits glutamate release by suppressing Ca_v_3.2 activity [24]. Thus, it is possible that the loss of I_h_ channel activity hyperpolarizes the RMP of ACs to a small window (−70mV to −60 mV) in which Cac channels are active. If this is true, then we should be able to suppress Cac activity and subsequent rhythmic depolarization in photoreceptors by changing AC RMP. Previous recordings in larval muscle fibers showed that expression of the sodium channel NaChBac evoked robust voltage-gated inward currents that began to activate at approximately −60 mV and peak at approximately −30 mV [43]. Therefore, we genetically depolarized the RMP of ACs by expressing NaChBac [43]. Interestingly, its expression in ACs but not in L1L2 neurons suppressed rhythmic depolarization in *I*_*h*_ mutant photoreceptors (Fig. 9C). However, both expression of dORK^ΔC^ and NaChBac in ACs failed to trigger rhythmic depolarization in wild-type flies (Fig. 8C and Fig. 9C). Taken together, these findings suggest that rhythmic depolarization in *I*_*h*_ mutant photoreceptors is due to changes in the RMP that relieve Cac inactivation in ACs.

### EKAR Receives Synaptic Glutamate Output from ACs

**Fig 10:**
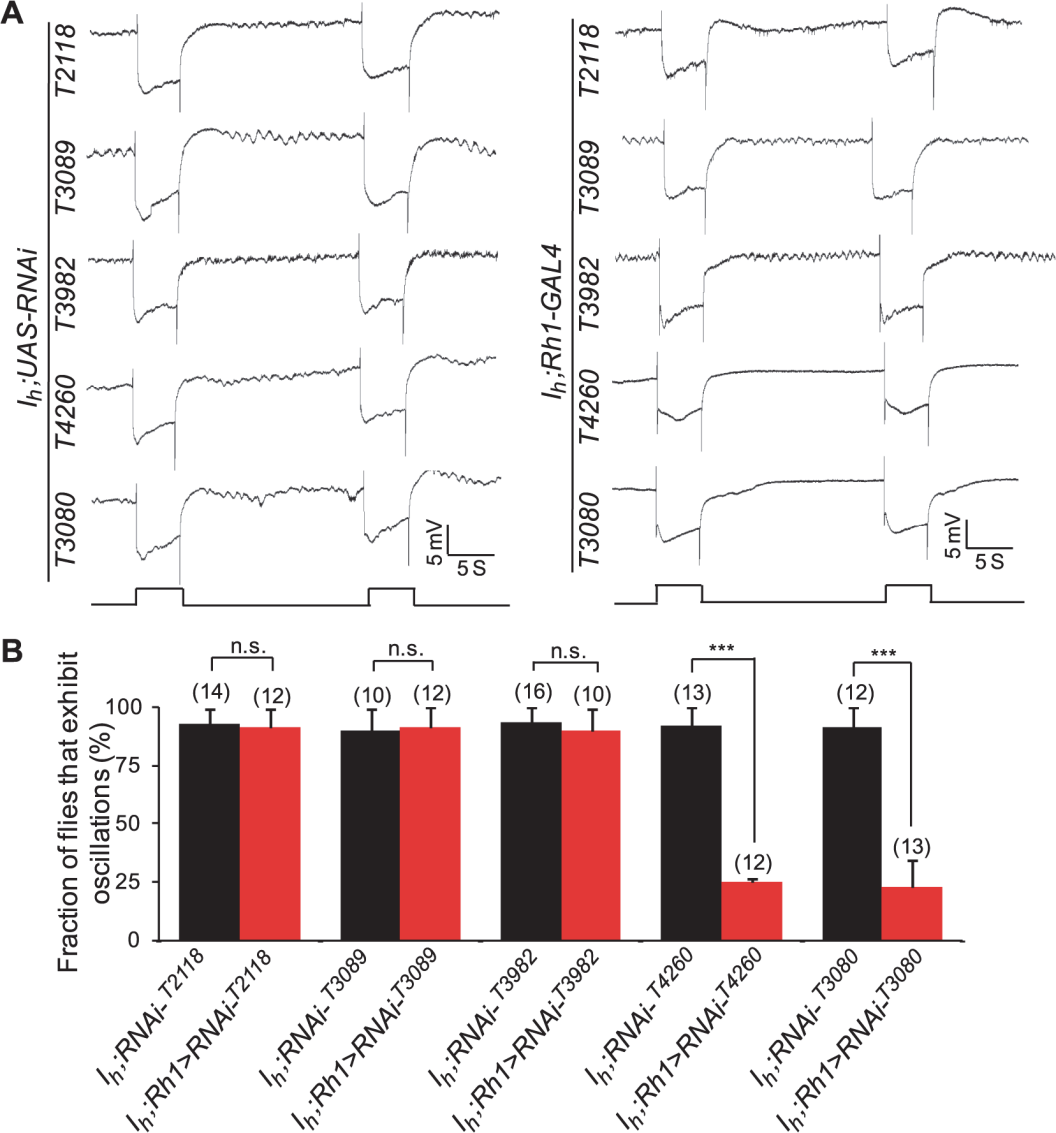
Identification of glutamate receptor that mediates retrograde glutamate signaling from ACs to photoreceptors. (**A**) ERG traces of flies with iGluR depletion in photoreceptors. Photoreceptor-specific Rh1-GAL4 was used for iGluR screening. (**B**) Fractions of flies exhibit ERG oscillation phenotype and the numbers of recorded flies are presented.

To identify the glutamate receptor that mediates retrograde glutamate signaling from ACs to photoreceptors, we screened 15 known ionotropic glutamate receptor (iGluR) subunits, including three conserved classes (kainate, AMPA, and NMDA types) of cation iGluRs and one chloride channel (GluClα) (Table 1) [20]. Interestingly, knockdown of ekar (CG9935, Gene ID: 43806) in photoreceptors by two individual RNAi lines, THU3080 and THU4260, suppressed ERG baseline oscillations in *I*_*h*_ mutant flies (Fig. 10). However, knockdown of other glutamate receptors in photoreceptors did not show any inhibition effects (Fig. 10).

**Table 1:** RNAi lines used for iGluR screening.

CG Number	Name	Gene ID	Locus	RNAi Line
**AMPA**				
CG8442	*GluRIA*	38742	65C1	THU2683
CG4481	*GluRIB*	44484	67A4–67A6	v106269, v42890
**NMDA**				
CG33513	*Nmdar2*	31107	2B1	v12189, v3196
CG2902	*Nmdar1*	3345094	83A6–83A7	THU2118
**GluClα**				
CG7535	*glc*	42350	92B1–92B2	v105754
**Kainate**				
CG6992	*GluRIIA*	33788	25E6–25E6	THU2659
CG7234	*GluRIIB*	33789	25E6–25E6	THU3089
CG4226	*GluRIIC*	33275	21E2–21E2	THU2049
CG18039	*GluRIID*	44483	92F4–92F4	THU2151
CG31201	*GluRIID*	318623	92F4–92F4	THU3986
CG8681	*clumsy*	35394	39B2–39B3	v105870, v1478
CG3822		42473	93A2–93A2	THU3982
CG5621		42476	93A2–93A3	THU3979
CG9935	*ekar*	43806	102D1–102D1	THU3080, THU4260
CG11155		43822	102F8–102F8	THU3285

**Fig 11:**
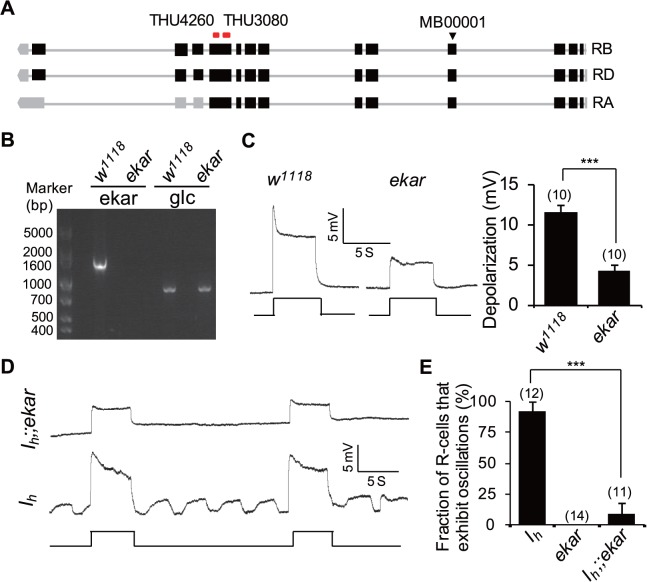
The kainate receptor EKAR receives the retrograde glutamate signal in photoreceptor terminals. (**A**) Annotated transcriptions of the *ekar* gene. The transposon insertion site of *Mi{ET1}CG9935* ^*MB00001*^ is indicated with a triangle. Recognized sites of two RNAi lines are labeled at the top. (**B**) RT-PCR analysis reveals an absence of ekar mRNA in *Mi{ET1}CG9935* ^*MB00001*^ line. The *Glc* gene is used as a positive control. (**C**) Intracellular recordings of light responses in wild-type and *ekar* mutant photoreceptors. Quantification of light-induced depolarization is presented in the right panel. For each genotype, ten photoreceptors from ten flies were measured, and the data are presented as mean ± SEM. (**D**) Intracellular recordings of *I*_*h*_;;*ekar* photoreceptors show that *ekar* mutation suppresses rhythmic depolarization in *I*_*h*_ mutant flies. (**E**) The fractions of photoreceptors exhibiting the oscillation phenotype are presented. The numbers of recorded photoreceptors are listed.

We also obtained an *ekar* mutant allele, *Mi{ET1}CG9935* ^*MB00001*^ [44], that does not produce ekar mRNA (Fig. 11A,B). Intracellular recordings revealed that light-induced depolarization of photoreceptors was significantly reduced in *ekar* mutant flies compared with wild-type flies (11.6±0.8 mV versus 4.3±0.8 mV, *p* < 0.001, Fig. 11C). However, EM images revealed normal rhabdomere structures in *ekar* mutants (S4A Fig.), and western blotting showed that *ekar* mutants express normal protein levels of phototransduction components (S4B Fig.). These observations suggest that EKAR might contribute to the light-evoked depolarization of photoreceptors. To further validate the role of EKAR in mediating the retrograde glutamate signal, we generated *I*_*h*_;;*ekar* double mutant flies. Intracellular recordings revealed that *ekar* mutation suppressed rhythmic depolarization in *I*_*h*_ mutant photoreceptors (Fig. 11D,E). Given that rhythmic depolarization in *I*_*h*_ mutant photoreceptors was independent of phototransduction cascades activation (Fig. 3D), the suppression of rhythmic depolarization in *I*_*h*_;;*ekar* double mutant flies indicates that the kainate receptor EKAR receives synaptic glutamate output from ACs.

### Feedback Regulation Is Critical for Visual Signal Transmission and Motion Detection in Dim Light Conditions

To explore the potential role of this feedback regulation, we first examined whether feedback regulation is required for photoreceptor survival. EM images revealed normal rhabdomere structure in 14-day-old *I*_*h*_ and *ekar* mutant flies raised under regular light cycles (12 h light/12 h dark) or in constant darkness (S5 Fig.). These observations suggest that feedback regulation from ACs to photoreceptor terminals is not essential for photoreceptor survival.

**Fig 12:**
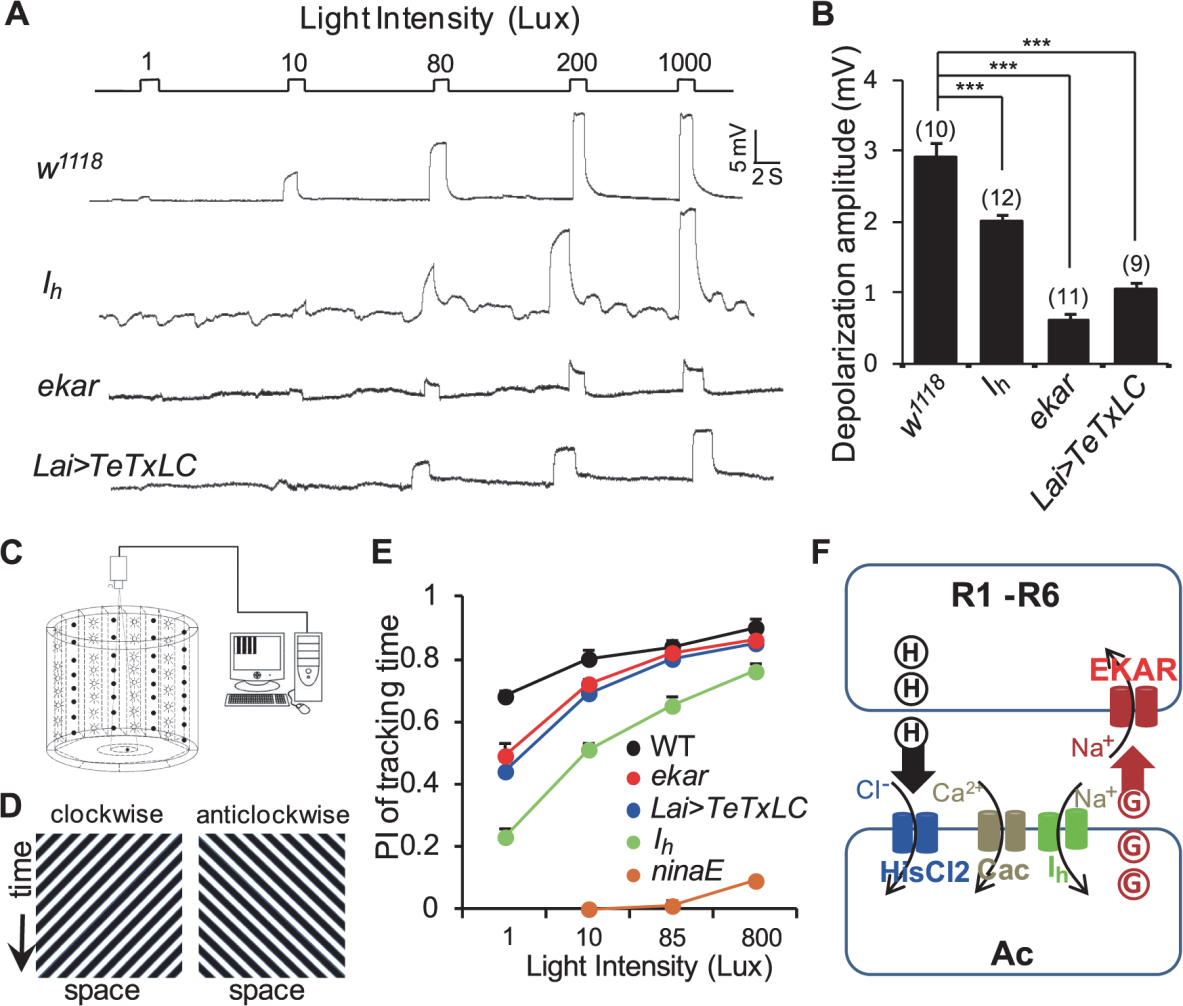
Feedback regulation facilitates visual signal transmission and motion detection in dim light conditions. (**A**) Intracellular recordings show photoreceptor responses to a series of 1.5-s light pulses with increasing intensity in each genotype. (**B**) Quantification of 10 Lux light-induced depolarization in each genotype. The numbers of recorded photoreceptors are listed for each genotype, and data are presented as mean ± SEM. (**C**) Schematic representation of the experimental apparatus. Only half of the LEDs are displayed. (**D**) Full-field stimuli (180°/s corresponding to a temporal frequency of 4 Hz) used in all behavioral experiments. The space-time diagrams illustrate the luminance patterns displayed to the fly in the arena. (**E**) Performance index of tracking time. More than 15 flies were examined for each genotype and condition, and the data are presented as mean ± SEM. (**F**) Model of feedback regulation from ACs to photoreceptors.

We next performed intracellular recordings to examine photoreceptors’ excitability in response to various light intensity stimulations. The results showed that photoreceptors underwent light-induced depolarization in a light intensity-dependent manner (Fig. 12A,B). 10 Lux light stimulation evoked a 2.91 ± 0.19 mV depolarization in wild-type photoreceptors and a significantly reduced depolarization in *I*_*h*_ mutant photoreceptors (2.01 ± 0.11 mV versus 2.91 ± 0.19 mV; *p* < 0.05, Fig. 12A,B), which was indistinguishable with rhythmic depolarization without light stimulation in *I*_*h*_ mutant photoreceptors (Fig. 12A). In contrast, 10 Lux light stimulation triggered a significantly reduced depolarization in *ekar* mutant photoreceptors (0.63 ± 0.10 mV versus 2.91 ± 0.19 mV; *p* < 0.001) and in *Lai-GAL4/UAS-TeTxLC* photoreceptors (1.04 ± 0.10 mV versus 2.91 ± 0.19 mV; *p* < 0.001) (Fig. 12A,B). These observations indicate that feedback regulation from ACs to photoreceptor terminals facilitates photoreceptor excitability and helps maintain light sensitivity in presence of ambient light.

To investigate the potential role of this feedback regulation in visual behavior, we assessed the flies’ optomotor responses under various light conditions. We placed single flies on a circular platform and examined their ability to track moving light patterns (Fig. 12C,D). With high-intensity moving light patterns (85 and 800 Lux), *I*_*h*_ mutant flies but not *ekar* mutant or *Lai-GAL4/UAS-TeTxLC* flies exhibited a reduced ability to track moving patterns (Fig. 12E). However, with low-intensity moving light patterns (1 and 10 Lux), *I*_*h*_ mutant flies, *ekar* mutant, and *Lai-GAL4/UAS-TeTxLC* flies were less able to track moving patterns (Fig. 12E). These findings demonstrate that feedback regulation from ACs to photoreceptor terminals enhances the flies’ optomotor response in dim light conditions, whereas uncontrolled feedback regulation also disturbs motion detection in ambient light conditions (Fig. 12E).

Based on these results, we propose a feedback regulation model from ACs to photoreceptors (Fig. 12F). Photoreceptors synthesize the inhibitory neurotransmitter histamine, which is released upon light stimulation [12,45]. Thus histamine hyperpolarizes ACs by opening HisCl 2 channels [13,46,47]. Hyperpolarization of ACs activates I_h_ channels, which depolarizes AC RMP and limits Cac channel activity. Without I_h_ channels in ACs, Cac channels are activated, resulting in Ca^2+^ influx and subsequent glutamate release from ACs. EKAR is expressed in photoreceptor terminals and depolarizes photoreceptors upon receiving the retrograde glutamate released from ACs.

## Discussion

### I_h_ Channels Regulate Retrograde Glutamate Release by Modulating Cac Activity

It has been shown I_h_ channels are expressed in several classes of interneurons that exhibit spontaneous firing activity and provide tonic inhibition to principal neurons, thus contributing to the regulation of firing frequency and excitability [48]. In this study, we revealed that loss of I_h_ channels in ACs results in rhythmic depolarization in photoreceptors. This phenotype was suppressed by either blocking neurotransmitter release or impairing synaptic glutamate output from ACs. Our studies provide solid evidence that feedback regulation from ACs to photoreceptors is regulated by I_h_ channels. Although expression of I_h_ channels in L1 and L2 neurons failed to suppress rhythmic depolarization in *I*_*h*_ mutant photoreceptors, L1/ L2 neuron-expressed I_h_ channels may also contribute to feedback regulation, as knockdown of I_h_ channels using recombinant Lai-GAL4 and L1L2-GAL4 led to enhanced ERG baseline oscillations compared with Lai-GAL4 alone. These findings are consistent with previous morphological studies showing that outer photoreceptor terminals also directly receive feedback inputs from L2 neurons [6–8].

Low-threshold Ca^2+^ channels are expressed in a variety of tissues such as the brain, heart, smooth muscle, kidney, and various endocrine glands [49]. These channels play important roles in controlling intracellular Ca^2+^ levels, modulating neuronal excitability, and regulating hormone and neurotransmitters secretion [50]. Here, we show that *cac* mutant flies suppressed glutamate release and subsequent rhythmic depolarization in *I*_*h*_ mutant photoreceptors. We further showed that rhythmic depolarization in *I*_*h*_ mutant photoreceptors were suppressed by changing the RMP of ACs. Our results suggest that HCN channels depolarize the RMP, thereby restricting Ca^2+^ entry via Cac channels and preventing glutamate release. Therefore, glutamate release is enhanced in *I*_*h*_ mutants, which causes rhythmic depolarization of photoreceptors. In addition, we showed that the released glutamate may induce long-lasting depolarization by opening of EKAR, which may contribute to the slow repolarization at the end of light stimulation in *I*_*h*_ mutant photoreceptors. A recent study reports that HCN1 channels localize in the active zone of mature asymmetric synaptic terminals and inhibit synaptic glutamate release by suppressing the activity of low-threshold voltage-gated T-type Ca^2+^ channels [24]. Thus, this form of regulation might be a common mechanism by which these channels modulate neuronal excitability.

### EKARs Receive Retrograde Glutamate Signals in Photoreceptor Terminals

Intracellular recordings from wild-type and *shibire* ^*TS*^ mutant flies reveal that cessation of all synaptic feedback to photoreceptors results in a 10–15 mV hyperpolarization shift upon light stimulation [14], suggesting that feedback regulation depolarizes photoreceptors upon light stimulation. However, the receptors that receive the excitatory neurotransmitter are still unknown. Our findings indicate that the EKAR receptor receives the glutamate signal in photoreceptor terminals: both knockdown of EKAR in photoreceptors and mutation of *ekar* suppressed rhythmic depolarization in *I*_*h*_ mutant photoreceptors. This result is supported by the previous microarray data, which showed that EKAR is highly expressed in the eye [51]. Our intracellular recording result also revealed that light-induced depolarization of photoreceptors was significantly reduced in *ekar* mutant flies compared with wild-type flies. The recordings further showed that *I*_*h*_ mutant photoreceptors undergo rhythmic depolarization with slow rise and decay times, which is consistent with the physiological properties of kainate receptors that mediate postsynaptic depolarization with slow rise and decay time [52]. Taken together, these results indicate that EKAR is expressed in photoreceptor terminals and depolarizes photoreceptors upon light stimulation.

### Feedback Regulation from ACs to Photoreceptors Helps Maintain Light Sensitivity in the Presence of Ambient Light

In vertebrates, the synaptic input from horizontal cells to cone cells contributes to many visual functions including the formation of center-surround receptive fields, retinal synchronization, and light adaptation [53–55]. Fly ACs are structurally equivalent to horizontal cell in vertebrates. A recent study showed that silencing ACs reduces optomotor responses to regressive rotation stimulation, whereas activation of ACs leads to slightly decreased responses to high and low contrast stimulations [31]. Although ACs project most of their synapses to epithelial glia, they also form direct feedback synapses to photoreceptor axons [6]. In this study, we show that feedback regulation from ACs to photoreceptors improves ambient light-induced visual signal transmission and motion detection under dim light conditions, which might be important for fly activity at dawn and dusk. Conversely, uncontrolled feedback regulation in *I*_*h*_ mutant flies impairs visual signal transmission and motion detection in ambient light conditions, suggesting that feedback regulation is strictly modulated. In this study, we showed that *I*_*h*_ mutants exhibit a significantly reduced ability in tracking the moving patterns with high light intensity. Since L1 and L2 neuron play essential roles in normal motion vision [31,56], loss of I_h_ channels in L1 and L2 neurons may contribute to this reduced ability in motion detection.

In *Drosophila* photoreceptors, the rapid termination of photoresponse in *Drosophila* is thought to be achieved by fast deactivation of rhodopsin and calcium-mediated intrinsic feedbacks [57,58]. In addition, a slow termination of photoresponse has been reported in mutants with blocked photoreceptor transmission [59], suggesting a retrograde regulation is likely to contribute to the termination speed. In this study, Our ERG and intracellular recordings showed that blocking neurotransmitter release from ACs resulted in slow repolarization at the end of light stimulation. Similar phenotypes are observed in flies with reduced glutamate signal output from ACs and *cac* mutant flies [60]. Given that most of mutants with slow termination phenotype did not undergo rhythmic depolarization [57–59,61–64], the slower depolarization phenotype is not sufficient to trigger the rhythmic depolarization. These observations suggest that feedback regulation from ACs is essential for the rapid repolarization of photoreceptors at the end of light stimulation. However, repolarization speed is normal in *ekar* mutant flies. Because ACs form most of feedback synapses to epithelial glia [6]. Rapid repolarization of photoreceptors might be regulated by glia cells. The mechanism that facilitates rapid repolarization at the end of light stimulation need to be further investigated.

In summary, our studies reveal the molecular mechanism and physiological roles of feedback regulation from ACs to photoreceptors. This might represent a general mechanism by which feedback regulation modulates synaptic transmission and facilitates neural circuit excitability and network information processing.

## Materials and Methods

### Fly Stock

Transposon *piggyBac* insertion flies *I*_*h*_^*f03355*^ and *I*_*h*_^*f01485*^ [25,26] were obtained from Harvard Medical School. The mutant alleles used for other genes in this work are *Cac* ^*H18*^ [65], *PBac{WHr}Ca-α1T* ^*del*^ [42], and *Mi{ET1}CG9935* ^*MB00001*^ [44,66]. UAS-RNAi lines were ordered from Vienna Drosophila RNAi Center and Tsinghua RNAi Stock Centre. *L1-GAL4*, *L2-GAL4*, and *L1/L2-GAL4* lines [33,34] were obtained from Dr. Jens Rister, and the split *Lai-GAL4* line (*R92A10AD attP40; R17D06DBD attp2*) [31] was provided by Dr. Aljoscha Nern. Other lines used in this work were obtained from the Bloomington Stock Center. The wild-type flies used in this study were *w* ^*1118*^. All flies were maintained in standard medium at 25°C, with 60%–80% relative humidity. Less than 3-day-old flies were used for ERG recording, and 3-day-old flies were used in optomotor response assays. In all experiments, an equal number of male and female flies were used. Full genotypes of samples shown in main figure panels are provided in S1 Table.

### Antibodies

Anti-I_h_ antibodies were generated in rabbits against a purified glutathione S-transferase fusion fragment (aa332–618) of I_h_-PK protein and generated by GenScript (Nanjing, China). An affinity column, generated by coupling a MBP-I_h_ fragment (aa332–618) to Sepharose 4B, was used to purify the antibody. The sources of other antibodies were rabbit anti-TRP [67], rabbit anti-Arr2 [61], rabbit anti-INAD [63], anti-PLC antibodies [68], anti-GFP (Abcam), and mouse anti-24B10 [69] and anti-Rh1 (4C5) (DSHB).

### Electrophysiological Recordings

Electroretinogram (ERG) recordings were conducted at 25°C as previously described [70]. Less than 3-day-old flies were collected, immobilized with strips of tape and put in darkness for 5 min for adaptation. Two glass microelectrodes were filled with Ringer’s solution and placed on the thorax and compound eye. Flies were stimulated with 5-s light pulses (4000 Lux) every 25 s using a Newport light projector. For each fly, ERG recording lasted for more than 100 s. The signal was amplified and recorded using a Warner IE210 Intracellular Electrometer. In ERG and intracellular recording without light stimulation, more than five continuous depolarizations with amplitude >1 mV was defined as an ERG baseline oscillation phenotype. The fraction of flies that exhibited ERG oscillation was quantified and presented in the figures.

Intracellular recordings were performed as described previously [63]. Briefly, flies were fixed with strips of tape, and a small opening was made on surface of the eye using fine tweezers. A low resistance (>30 MΩ) glass microelectrode filled with 2 M KCl was gradually inserted into the opening until light-induced membrane depolarization was observed. A reference electrode was filled with Ringer’s solution and placed inside the eye at the retina layer. The signal was amplified and recorded using a Warner IE210 Intracellular Electrometer. The fractions of photoreceptors exhibiting rhythmic depolarization in each genotype were calculated. To quantify the amplitudes of light responses, 10 photoreceptors from 10 flies were measured for each genotype, and the mean±SEM was calculated and showed in figures.

### Western Blotting

Western blotting was carried out as previously described [71]. Fly heads were homogenized in SDS-sample buffer. The proteins were fractionated by SDS-PAGE and transferred to PVDF membranes (Pall) in Tris-glycine buffer. After blocking, the blots were probed with anti-Rh1 antibody (1:3,000 dilution), rabbit anti-Arr2 antibody (1:1,000 dilution), rabbit anti-INAD antibody (1:1,000 dilution), anti-I_h_ antibody (1:200), rabbit anti-TRP antibody (1:1,000), anti-PLC antibody (1:1,000) at RT for 2 h. After three washes with PBS, the blots were subsequently probed with either anti-rabbit or mouse IgG-peroxidase conjugate (GE Healthcare) at RT for 1 h, and the signals were detected using ECL reagents (Amersham Biosciences)

### Retina and Lamina Isolation

To separate the retina and lamina from the brain for western blotting analysis, fly heads were cut and immersed in 100% ethanol for 2 h before the retina and lamina were carefully dissected from the brain. Separated tissues (retina and lamina, the head without retina and lamina) were homogenized in SDS-sample buffer. To isolate the retina and lamina for RT-PCR analysis, they were dissected carefully, and the mRNA was extracted from the separated tissues using TRIzol reagent (Invitrogen).

### Immunostaining

Section staining was performed as previously described [61]. Fly heads were fixed with 4% paraformaldehyde. After three washes, the fly heads were dehydrated with acetone and embedded in LR White resin, and 1-μm cross-sections were cut across the top half of the eye. The sections were incubated in diluted primary antiserum (Rh1, 1:200; INAD, 1:400; TRP, 1:400) at room temperature for 1 h. After three times of washing in PBS, sections were incubated with diluted secondary antibodies at room temperature for 1 h. The stained sections were examined under a ZEISS Axio observer A1 microscope.

Whole-head staining was performed to locate endogenous I_h_ channel in wild-type adult flies. Fly heads were dissected in PBS buffer and fixed with 4% paraformaldehyde in PBS buffer. After fixation, the heads were double labeled with diluted primary antiserum (anti-I_h_ antibodie 1:50 and 24B10 1:100 or anti-GFP 1:200). After three washes in PBS buffer, fly heads were incubated with diluted secondary antibodies at room temperature for 1 h [71]. After three additional PBS washes, the stained heads were examined under an LSM 700 confocal microscope.

### Electron Micrograph

EM was carried out as previously described [72]. Fly heads were fixed at 4°C for 12 h in 2.5% gluteraldehyde, 0.1 M sodium cacodylate (pH 7.2). After three washes with 0.1 M sodiumcacodylate, the heads were stained with 1% osmium tetroxide at room temperature for 1 h. After a standard ethanol dehydration series, the heads were immersed in propylene oxide for two 10-min washes before they were embedded with standard procedures. Thin sections (100 nm) were cut at the top 2/3 of retina to show ommatidia whereas cut at half the lamina to display cartridges. Sections were collected on Cu support grids and stained with uranyl acetate for 8 min, followed by 5 min with lead citrate. Micrographs were taken at 80 kV on a Hitachi-7650 transmission EM.

### Optomotor Responses Assay

Fly optomotor responses were tested as previously described [73]. Briefly, 3-day-old flies were collected and their wings were cut off. After recovering for more than 24 h in a 12-h light/12-h dark cycle, a single fly was placed on a circular platform for the optomotor response test. The platform is surrounded with a water-filled moat to prevent the fly from escaping, and the moat was surrounded with a panoramic LED display that controlled by LED Studio software (Shenzhen Sinorad Medical Electronics). Bright and dark stripes were used to generate a clockwise motion light for 90 s followed by an anti- clockwise motion light for another 90 s (180°/s corresponding to a temporal frequency of 4 Hz). The walking traces of flies were recorded by a camera (WV-BP330, Panasonic System Networks), and the data were analyzed in MATLAB (Mathworks). The flies’ optomotor responses were quantified by the performance index of tracking time (PITT). The PITT is defined as (P_tracking time_—P_un-tracking time_)/ (P_tracking time_ + P_un-tracking time_). The probabilities of fly movement in the platform in accordance with LED rotating direction or not are defined as tracking or un-tracking, respectively. The male and female flies were used alternately.

### Statistics

Statistical analysis was done by using MS Excel. The numerical data used in all figures are included in S1 Data. For quantitative data of ERG and intracellular recordings, fraction of flies or photoreceptors that exhibit oscillations and standard error of rate are shown. Fisher’s exact probability tests were used to compare genotypes. For statistical analysis of depolarization amplitudes and decay time of depolarization in Figs. 2C, 8B, 8D, 11C, and 12B, data are presented as mean ± SEM. Two-tailed Student’s *t* tests were used to compare genotypes. Significance was classified as follows: *, *p* ≤ 0.05; **, *p* < 0.01; ***, *p* < 0.001; n.s. *p* > 0.05.

## Supporting Information

S1 Data Excel sheet containing the numerical data and statistical analysis for Figs. 1C, 2B, 2C, 3D, 5C, 7B, 7D, 8B, 8C, 8D, 9A, 9B, 9C, 10B, 11C, 11E, 12B, 12E; and S1B, S2C, S3A, and S3B Figs. (XLSX)

S1 Fig Characterization of ERG baseline oscillation phenotype in *I*_h_ mutant flies. A) *I*_*h*_ mutant lines exhibit ERG baseline oscillation with variable frequency and depolarization. B) ERG traces from a single *I*_*h*_ mutant fly at different time points after light stimulation. The number represents the time after light stimulation. The time scale bar represents 5 s, and the depolarization scale bars are 5 mV. The averaged frequency and amplitude of depolarization at each time point are presented in the right panel.
(EPS)

S2 Fig Depletion of long isoforms of I_h_ channels in the eye show normal ERG responses. A) Expression levels of I_h_ channels in the flies with depletion of long isoforms of I_h_ channels using *UAS-I*_*h*_-*RNAi-v110274* driven by anatomically restricted GAL4 drivers. A single copy of the GAL4 driver was used for each GAL4 line. Each lane was loaded with two fly heads. The I_h_ channel bands are indicated with arrows. Note that only long isoforms of Ih channels (170 and 125 kDa), but not short isoforms (73 and 71 kDa), were suppressed in *elav-GAL4;UAS-I*_*h*_-*RNAi* ^*v110274*^ flies. B) ERG traces of flies with long-isoform of I_h_ channel depletion. C) The fractions of flies that exhibited ERG oscillation phenotype. The numbers recorded for each genotype are listed.
(EPS)

S3 Fig Blocking synaptic glutamate release from ACs suppresses the rhythmic depolarization in *Ih* mutant photoreceptors. A) ERG traces of *Ih* mutant flies with TeTxLC expressing using *L1L2-GAL4* and *Lai-GAL4* drivers. An ERG trace of flies expressing TeTxLC in wild-type ACs is also presented. The fractions of flies exhibiting the ERG oscillation phenotype are presented in the right panel, and the numbers of recorded flies are listed. B) ERG traces of *Ih* mutant flies expressing *UAS-vGluT-RNAi* under different drivers. The fractions of flies exhibiting the ERG oscillation phenotype are presented in the right panel, and the numbers of recorded flies for each genotype are listed.
(EPS)

S4 Fig *ekar* mutant flies exhibit normal rhabdomeral structure and normal protein levels of phototransduction components. A) EM images show normal rhabdomeral structure in 1-day-old *ekar/+ and ekar* mutant flies. B) Western blotting shows normal protein levels of phototransduction components in *ekar* mutant flies.
(EPS)

S5 Fig *I*_h_ mutant and *ekar* mutant flies did not undergo retinal degeneration. EM iamges reveal normal rhabdomeral structure in 14-day-old *I*_*h*_ mutants and *ekar* mutants raised under either regular light cycles (12 h light/12 h dark) or in constant dark condition.
(EPS)

S1 Table Full genotypes of flies used in this study. (XLSX)

## Abbreviations:

AC: amacrine cell
Cac: cacophony
Ca_v_3.2: low-threshold voltage-gated T-type Ca^2+^ channel
EKAR: eye-enriched kainate receptor
EM: electron microscopy
ERG: electroretinogram
iGluR: ionotropic glutamate receptor
I_h_ channel: hyperpolarization-activated cyclic nucleotide-gated (HCN) channel
RMP: resting membrane potential
RT-PCR: reverse transcription polymerase chain reaction
TeTxLC: tetanus toxin light chain
TRP: transient receptor potential
VGCC: voltage-gated Ca^2+^ channel
vGluT: vesicular glutamate transporter
